# Impact of Definitive Radiotherapy and Surgical Debulking on Treatment Outcome and Prognosis for Locally Advanced Masaoka-Koga stage III Thymoma

**DOI:** 10.1038/s41598-020-58692-2

**Published:** 2020-02-03

**Authors:** Chengcheng Fan, Hong Ge, Shaokai Zhang, Wenqun Xing, Ke Ye, Yan Zheng, Haibo Sun, Hao Wang, Xiaoli Zheng, Ruiyun Zhang, Meiling Liu

**Affiliations:** 1 Department of Radiation Oncology, The Affiliated Cancer Hospital of Zhengzhou University, Henan Cancer Hospital, Zhengzhou, China; 2 Department of Cancer Epidemiology, Henan Office for Cancer Control and Research, The Affiliated Cancer Hospital of Zhengzhou University, Henan Cancer Hospital, Zhengzhou, China; 3 Department of Thoracic Surgery, The Affiliated Cancer Hospital of Zhengzhou University, Henan Cancer Hospital, Zhengzhou, China

**Keywords:** Cancer, Medical research, Oncology

## Abstract

The role of definitive radiotherapy (dRT) and debulking surgery (DS) for patients with locally advanced, unresectable, Masaoka-Koga stage III thymomas was not well studied. Unresectable tumor refers to tumor that could not be completely resected because of invasion of surrounding organs. Consecutive patients with unresectable stage III thymomas between 2000 and 2017 were reviewed. According to the treatment intent and radiation dose, patients were categorized into a dRT group and a non-dRT group. The former group included patients who received radiotherapy at doses ≥ 54 Gy after DS or biopsy. The latter group included patients who did not receive radiotherapy and those who received a radiation dose < 54 Gy. A total of 82 patients were included. Compared with non-dRT, dRT significantly improved 5-year overall survival (OS, P = 0.003), progression-free survival (PFS, P = 0.008), and freedom from locoregional failure (FFLF, P < 0.001). Compared with biopsy alone, DS did not improve OS, PFS, FFLF. On multivariate analysis, dRT was an independent prognostic factor for OS (hazard ratio [HR]: 2.37, P = 0.024), PFS (HR: 2.40, P = 0.004), and FFLF (HR: 3.83, P = 0.001). In conclusion, dRT was an effective and beneficial treatment for patients with unresectable Masaoka-Koga stage III thymoma.

## Introduction

Surgery is the cornerstone of the treatment of thymic malignancies. After complete surgical resection of the tumor, most patients have a good prognosis^1–4^. However, complete tumor resection is often difficult to achieve in the case of Masaoka-Koga stage III thymomas. Surgeons often recommend thoracotomy with or without prior neoadjuvant treatment to maximize the chances of complete tumor resection. If thoracotomy confirms that complete resection is not possible, then surgeons must decide whether or not debulking surgery (DS) should be carried out. Current evidence on patient survival after DS for thymoma is contradictory, but most studies do not show any survival benefit of DS^1–3,5–20^.

Radiotherapy is often administered after surgery for locally advanced thymoma, but the radiation dose varies according to the treatment intent. In the case of tumors that have been completely resected, radiotherapy is administered as an adjuvant therapy at a moderate dose of 45–50 Gy^21,22^. In the case of unresectable or incompletely resected tumors, radiotherapy may lead to long-term tumor control or even complete remission. Thus, radiotherapy with the intention to cure and doses ≥ 54 Gy is considered definitive radiotherapy (dRT), as proposed by the International Thymic Malignancy Interest Group (ITMIG)^23^. As a local treatment, dRT with curative intent has been proved to play an important role in other locally advanced, unresectable thoracic malignancies such as non-small cell lung cancer and esophageal cancer^24,25^. However, to our knowledge, very rare studies have attempted to evaluate the effects of radiotherapy for locally advanced, unresectable thymic malignancies, and the role of dRT in this condition remains unclear^26–28^. In the current study, unresectable thymoma refers to tumor that could not be completely resected because of invasion of surrounding organs. We focused on the role of dRT and surgical debulking in the treatment of such patients.

## Methods

### Patient selection

We collected the data of consecutive patients with primary thymic epithelial malignancies treated between January 2000 and December 2017 in our institution. According to the Masaoka-Koga classification system^29^, the data of patients with unresectable stage III tumors, regardless of whether they had undergone DS, were extracted for the current analysis. All patients included in this study were confirmed to have thymoma via the histological examination of a postoperative pathological specimen or a biopsy specimen. Histological subtypes reported based on the Muller-Hermelink classification were converted to the corresponding WHO histological classification, consisting of types A, AB, B1, B2, and B3. The predominant subtype was used for analysis if more than one subtype was present.

### Treatment details

The surgical notes were carefully reviewed to determine the completeness of tumor resection. Median sternotomy had been conducted to determine whether a tumor could be completely resected. After the surgical exploration, some patients whose tumors could not be completely resected underwent DS, while others underwent surgical biopsy. The most common reason for the incomplete resection was great vessel invasion, which was defined as the invasion of at least one of the following vessels: brachiocephalic vessels, superior vena cava, hilar pulmonary vessels, aorta, arch vessels, and main pulmonary artery. DS was defined as the gross tumor volume removal of more than 90% but not complete resection, regardless of whether the invaded tissue was resected or not. Clips were placed at the site of the residual tumor after DS to facilitate the identification of the target volume for postoperative radiotherapy.

According to the treatment intent and radiation dose delivered, the patients in the current study were categorized into a dRT group and a non-dRT group. The former group included patients who received radiotherapy at doses ≥ 54 Gy after DS or biopsy. The non-dRT group included patients who did not receive radiotherapy, and those who received a radiation dose < 54 Gy. The gross tumor and tumor bed were considered the target volume in patients who had undergone biopsy only and DS, respectively. The median prescribed dose was 60 Gy (range, 10–70 Gy) administered in 1.8–2 Gy daily fractions. Before 2008, patients were administered conventional radiotherapy with a 6- or 8-MV X-ray device, using an anteroposterior opposed field or angled anterior fields. Two anterior, wedged portals or off-cord, oblique, opposed portals were often used to provide a boost to the residual anterior mediastinal tumor. After 2008, patients were administered conformal radiotherapy and intensity-modulated radiotherapy with a three-dimensional (3D) planning system.

Neoadjuvant or adjuvant chemotherapy was administered in patients who underwent DS. Sequential chemoradiotherapy was administered in most patients who underwent biopsy only, and a small group of patients received concurrent chemoradiotherapy. The median cycle of chemotherapy was 3 (1–6 cycles). Platinum-based doublets or triplets regimens were used.

### Follow-up

Follow-up assessments consisting of a physical examination, abdominal ultrasonography, and chest computed tomography were performed every 3 months for the first 2 years after treatment, then every 6 months for the following 3 years, and annually thereafter. Treatment failure was classified into locoregional failure (LF) and distant metastasis (DM). The term LF encompassed local failure and regional failure. Local failure was defined as disease relapse within the tumor bed or enlargement of the gross tumor, while regional failure was defined as the appearance of new intrathoracic lesions in the mediastinum, pleura, diaphragm, or pericardium. DM was defined as disease relapse in any part of the body beyond the thorax or in the intrapulmonary nodules. Whenever tumor progression was suspected, we attempted to obtain histological or unequivocal radiological proof. Overall survival (OS) was defined as the time from the date of diagnosis to the date of death or of the final follow-up, and progression-free survival (PFS) was defined as the time from the date of diagnosis to disease progression or death. Freedom from LF (FFLF) was defined as the time from the date of diagnosis to local progression or regional recurrence, and freedom from DM (FFDM) was defined as the time from the date of diagnosis to DM.

### Statistical analysis

All analyses were conducted using SPSS, version 24.0 (SPSS, Chicago, IL, USA). The distribution of categorical variables was tested using the chi square test. The Kaplan–Meier method was used to estimate survival rates and the rates of freedom from treatment failure, and the log rank test was used to examine the differences between the groups. The Cox proportional hazards model was used to perform univariate and multivariate analyses for OS, PFS, FFLF, and FFDM. All variables in univariate analysis were included in multivariate analysis. A P value of less than 0.05 was considered statistically significant.

### Ethics approval statement

This study was approved by the Institutional Review Board of the Affiliated Cancer Hospital of Zhengzhou University. The study was carried out in accordance with relevant guidelines. Informed consent was obtained from all participants before treatment according to the institutional guidelines. All data were analyzed retrospectively and anonymously.

## Results

### Patient characteristics

A total of 82 patients who met the inclusion criteria were enrolled in this analysis. The demographic characteristics of patients according to treatment modality are presented in Table 1. The median follow-up time was 41 months (range, 5–166 months). The median OS and PFS of all patients were 65 months (range, 5–166 months) and 50 months (range, 2–155 months), respectively.

**Table 1:** Demographic characteristics of patients.

	Surgery			Radiotherapy		
	DS	Biopsy	*p* value	dRT	Non-dRT	*p* value
	(n = 36)	(n = 46)		(n = 54)	(n = 28)	
**Sex**			0.384			0.854
Male	23	25		32	16	
Female	13	21		22	12	
Age	49.9±10.5	50.4±15.5	0.868	49.9±13.2	50.7±14.1	0.807
**ECOG PS**			0.241			0.671
0	25	30		38	17	
1	10	10		12	8	
2	1	6		4	3	
**Tumor size (cm)**			0.254			0.346
≤7cm	17	16		30	19	
>7cm	19	30		24	9	
**WHO histology**			0.847			0.052
A	4	9		11	2	
AB	4	4		5	3	
B1	11	12		14	9	
B2	6	6		4	8	
B3	11	15		20	6	
**MG**			0.067			0.351
Yes	4	9		7	6	
No	42	27		47	22	
**Great-vessel invasion**			0.028			1
Yes	30	27		37	20	
No	6	19		17	8	
**Pericardial invasion**			0.38			0.489
Yes	21	22		30	13	
No	15	24		24	15	
**Lung invasion**			0.822			0.246
Yes	14	20		25	9	
No	22	26		29	19	
**Years**			0.845			0.163
2000–2010	18	24		31	11	
2011–2017	18	22		23	17	
**Chemotherapy**			0.001			0.353
Yes	10	30		24	16	
No	26	16		30	12	
**Surgery**						0.051
DS	36	0		22	14	
Surgical biopsy	0	22		19	3	
Aspiration biopsy	0	24		13	11	
**dRT**			0.486			
Yes	22	32		54	0	
No	14	14		0	28	
**Doses**			0.541			
>60 Gy	7	11		18	0	
=60 Gy	11	19		30	0	
≥54, <60 Gy	4	2		6	0	
<54, ≥10 Gy	7	5		0	12	
0 Gy	7	9		0	16	

Abbreviations: DS, debulking surgery; dRT, definitive radiotherapy; ECOG PS, Eastern Cooperative Oncology Group performance status; MG, myasthenia gravis; WHO, World Health Organization.

### Impact of dRT and DS on OS and PFS

**Figure 1:**
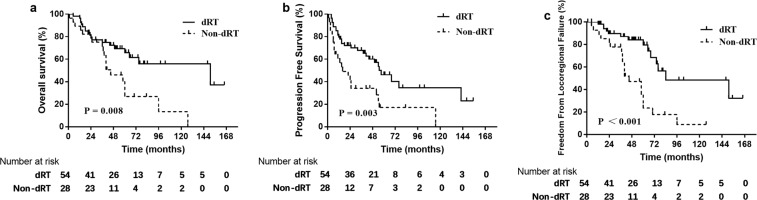
Kaplan–Meier curves depicting OS (**a**), PFS (**b**), and FFLF (**c**)in the dRT and non-dRT groups. *Abbreviations:* OS, overall survival; PFS, progression-free survival; FFLF, freedom from locoregional failure; dRT, definitive radiotherapy.

Patients who received dRT had improved OS and PFS compared with patients who did not receive dRT (Fig. 1a,b). The 5- and 10-year OS rates were significantly higher in the dRT group (65.7% and 55.8%, respectively) than in the non-dRT group (26.8% and 13.4%, respectively; P = 0.008). Additionally, the 5- and 10-year PFS rates were significantly higher in the dRT group (46.1% and 34.6%, respectively) than in the non-dRT group (17.0% and 0%, respectively; P = 0.003).

Compared with biopsy alone, DS did not improve the OS or PFS. The 5- and 10-year OS rates were 49.6% and 24.8%, respectively, among patients who underwent DS and 55.2% and 50.2%, respectively, among patients who underwent biopsy only (P = 0.921). Likewise, the 5- and 10-year PFS rates were similar in patients who underwent DS (27.0% and 13.5%, respectively) and those who underwent biopsy only (41.5% and 31.1%, respectively; P = 0.740).

No significant statistical difference was found in OS or PFS between patients who underwent both DS and dRT and those who did not. The 5- and 10-year OS rates were 63% and 31.5%, respectively, among patients who underwent both DS and dRT and 47.2% and 37.6%, respectively, among patients who did not undergo both DS and dRT (P = 0.175). The 5- and 10-year PFS rates were 37.3% and 37.3%, respectively, among patients who underwent both DS and dRT and 34.8% and 18%, respectively, among patients who did not undergo both DS and dRT (P = 0.149).

Table 2 showed that tumor size >7 cm (P = 0.014), B2 or B3 subtype (P = 0.002), absence of dRT (P = 0.008), and unimodal treatment (P = 0.038) were found to be associated with worse OS, and tumor size >7 cm (P = 0.019), B2 or B3 subtype (P = 0.004), and absence of dRT (P = 0.003) were found to be associated with worse PFS on univariate analysis. On multivariate analysis (Table 3), tumor size >7 cm (hazard ratio [HR]: 2.37, 95% confidence interval [CI]: 1.09–5.15, P = 0.029), B2 or B3 subtype (HR: 0.38, 95% CI: 0.19–0.74, P = 0.005), and absence of dRT (HR: 2.12, 95% CI: 1.10–4.06, P = 0.024) were associated with worse OS, while tumor size >7 cm (HR: 2.55, 95% CI: 1.29–5.04, P = 0.007) and absence of dRT (HR: 2.40, 95% CI: 1.33–4.38, P = 0.004) were associated with worse PFS.

**Table 2:** Results of univariate analysis for overall survival and progression-free survival.

Variable	OS			PFS		
	HR	95% CI	*P* Value	HR	95% CI	*P* Value
**Sex**						
Male	1			1		
Female	0.8	0.40–1.52	0.456	0.85	0.47–1.50	0.566
Age	1	0.99–1.04	0.233	1.02	0.99–1.04	0.171
**Tumor size**						
≤7cm	1			1		
>7cm	2.6	1.21–5.60	0.014	2.09	1.13–3.86	0.019
**Histology**						
B2/B3	1			1		
Others	0.4	0.18–0.69	0.002	0.39	0.22–0.71	0.004
**MG**						
Yes	1			1		
No	1	0.41–2.35	0.957	1.33	0.56–3.14	0.519
**Great-vessel invasion**						
Yes	1					
No	1.2	0.58–2.39	0.658	1.65	0.91–3.00	0.102
**Pericardial invasion**						
Yes	1			1		
No	0.9	0.47–1.71	0.742	0.99	0.57–1.75	0.984
**Lung invasion**						
Yes	1			1		
No	0.8	0.41–1.49	0.456	0.77	0.44–1.36	0.367
**Years**						
2000–2010	1			1		
2011–2017	0.7	0.33–1.30	0.23	0.79	0.44–1.43	0.436
**Chemotherapy**						
Yes	1			1		
No	1.4	0.73–2.68	0.317	0.99	0.57–1.75	0.987
**DS**						
Yes	1			1		
No	1	0.54–1.98	0.921	1.1	0.62–1.95	0.74
**dRT**						
Yes	1			1		
No	2.3	1.22–4.48	0.008	2.33	1.32–4.13	0.003
**Multimodal treatment**						
Yes	1			1		
No	4.8	1.04–4.74	0.038	1.45	0.80–2.63	0.222

Abbreviations: DS, debulking surgery; dRT, definitive radiotherapy; HR, hazard ratio; CI, confidence interval; MG, myasthenia gravis; OS, overall survival; PFS, progression-free survival.

**Table 3:** Results of multivariate analysis for overall survival and progression-free survival.

Variables	OS			PFS		
	HR	95% CI	*P* Value	HR	95% CI	*P* Value
**Tumor Size**						
≤7cm	1			1		
>7cm	2.4	1.09–5.15	0.029	2.6	1.29–5.04	0.007
**Histology**						
B2/B3	1			1		
Others	0.4	0.19–0.74	0.005	0.6	0.31–1.09	0.092
**dRT**						
Yes	1			1		
No	2.1	1.10–4.06	0.024	2.4	1.33–4.38	0.004

Abbreviations: dRT, definitive radiotherapy; OS, overall survival; PFS, progression-free survival; HR, hazard ratio; CI, confidence interval.

### Impact of dRT on treatment failure

Of the 82 patients, 45 (54.9%) showed tumor progression after treatment. LF and DM were identified in 18 and 11 patients, respectively, and concurrent LF and DM was identified in 16 patients. Of the 34 patients with LF, 5 had local failure, 16 had regional failure, and 13 had both local and regional failure. The most common sites of LF and DM were the pleura (26/34) and lung (11/27), respectively. Other sites of LF included the tumor bed or the primary tumor (18/34), pericardium (5/34), and diaphragm (2/34). Other DM sites included the bones (8/27), liver (4/27), extrathoracic lymph nodes (4/27), and brain (2/27). The median time to treatment failure was 15 months (range, 2–142 months).

Patients who received dRT had better FFLF than patients who did not (Fig. 1c). The 5- and 10-year FFLF rates were significantly higher in the dRT group (79.6% and 48.5%, respectively) than in the non-dRT group (23.6% and 8.8%, respectively; P < 0.001). However, the 5- and 10-year FFDM rates did not differ between the dRT group (73.8% and 47.4%, respectively) and non-dRT groups (60.7% and 22.7%, respectively; P = 0.419). Compared with biopsy only, DS did not improve FFLF (P = 0.803) or FFDM (P = 0.979).

Table 4 showed that tumor size >7 cm (P = 0.011), B2 or B3 subtype (P = 0.002), and absence of dRT (P < 0.001) were found to be associated with worse FFLF, and tumor size >7 cm (P = 0.014) and B2 or B3 subtype (P < 0.001) were found to be associated with worse FFDM on univariate analysis. On multivariate analysis (Table 5), tumor size >7 cm (HR: 5.63, 95% CI: 1.99–15.91, P = 0.001), great-vessel invasion (HR: 2.36, 95% CI: 1.09–5.12, P = 0.029), and absence of dRT (HR: 3.83, 95% CI: 1.76–8.31, P = 0.001) were associated with worse FFLF, and tumor size >7 cm (HR: 4.38, 95% CI: 1.79–15.01, P = 0.002), B2 or B3 subtype (HR: 0.14, 95% CI: 0.05–0.37, P < 0.001), and great-vessel invasion (HR: 4.09, 95% CI: 1.50–11.12, P = 0.006) were associated with worse FFDM.

**Table 4:** Results of univariate analysis for freedom from locoregional failure and freedom from distant metastasis.

Variable	FFLF			FFDM		
	HR	95% CI	*P* Value	HR	95% CI	*P* Value
**Sex**						
Male	1			1		
Female	1.2	0.58–2.31	0.671	0.8	0.38–1.84	0.661
Age	1	0.99–1.04	0.16	1	0.99–1.05	0.244
**Tumor size**						
≤7cm	1			1		
>7cm	3	1.28–7.01	0.011	3.2	1.26–8.06	0.014
**Histology**						
B2/B3	1			1		
Others	0.3	0.16–0.66	0.002	0.2	0.09–0.50	<0.001
**MG**						
Yes	1			1		
No	0.9	0.35–2.38	0.848	4.2	0.56–30.78	0.163
**Great-vessel invasion**						
Yes	1			1		
No	1.6	0.80–3.38	0.177	1.7	0.75–3.67	0.21
**Pericardial invasion**						
Yes	1			1		
No	0.8	0.40–1.62	0.547	1.6	0.72–3.38	0.257
**Lung invasion**						
Yes	1			1		
No	0.7	0.35–1.37	0.288	0.8	0.38–1.76	0.614
**Years**						
2000–2010	1			1		
2011–2017	1.2	0.57–2.34	0.681	0.6	0.24–1.31	0.185
**Chemotherapy**						
Yes	1			1		
No	0.9	0.42–1.72	0.66	1.4	0.65–2.95	0.406
**DS**						
Yes	1			1		
No	1.1	0.54–2.21	0.803	1	0.47–2.17	0.979
**dRT**						
Yes	1			1		
No	3.2	1.70–7.05	<0.001	1.4	0.63–3.03	0.419
**Multimodal treatment**						
Yes	1			1		
No	1.8	0.84–3.8	0.134	1.9	0.82–4.37	0.134

Abbreviations: DS, debulking surgery; dRT, definitive radiotherapy; HR, hazard ratio; CI, confidence interval; MG, myasthenia gravis; FFLF, freedom from locoregional failure; FFDM, freedom from distant metastasis.

**Table 5:** Results of multivariate analysis for freedom from locoregional failure and freedom from distant metastasis.

Variables	FFLF			FFDM		
	HR	95% CI	*P* Value	HR	95% CI	*P* Value
**Tumor Size**						
≤7cm	1			1		
>7cm	5.6	1.99–15.91	0.001	4.4	1.79–15.01	0.002
**Histology**						
B2/B3	1			1		
Others	0.5	0.20–1.05	0.064	0.1	0.05–0.37	<0.001
**Great vessel invasion**						
Yes	1			1		
No	2.4	1.09–5.12	0.029	4.1	1.50–11.12	0.006
**dRT**						
Yes	1			1		
No	3.8	1.76–8.31	0.001	1.2	0.48–2.98	0.691

*Abbreviations:* HR, hazard ratio; CI, confidence interval; FFLF, freedom from locoregional failure; FFDM, freedom from distant metastasis; dRT, definitive radiotherapy.

### Impact of radiation dose on treatment outcome

Among patients who received dRT, only 6 patients were delivered dose of ≥ 54 Gy but <60 Gy, and the 5-year OS rates for these 6 patients were not reached. The 5- and 10-year OS rates were 65% and 44.6%, respectively, among patients given dose of >60 Gy and 68.1% and 68.1%, respectively, among patients given dose of 60 Gy (P = 0.538). No significant statistical difference was found in PFS (P = 0.842) and FFLF (P = 0.729) rates between patients given dose of >60 Gy and 60 Gy.

## Discussion

The present study is the largest study ever to focus on the roles of dRT and DS in locally advanced, unresectable stage III thymoma. Conventionally, radiotherapy was administered to patients after thymoma resection. For patients with completely resected stage II or III tumors, postoperative radiotherapy was a controversial adjuvant therapy. Some small-sample retrospective studies reported that postoperative radiotherapy did not provide a survival benefit for patients with completely resected thymoma^22,30^. However, the results of a meta-analysis and a retrospective analysis of the ITMIG database demonstrated that postoperative radiotherapy is beneficial for Masaoka-Koga stage II and III patients with complete tumor resection^21,31^. However, the role of radiotherapy for Masaoka-Koga stage III thymoma that could not be completely resected was not well studied. Very few studies have reported the results of radiotherapy for unresectable thymic malignancies. Lin *et al*. reported the results of 27 patients with locally advanced Masaoka-Koga stage III and IVa thymic malignancies; they found that radiotherapy with doses >44 Gy significantly improved survival (P = 0.016)^26^. Chen *et al*. reported the results of 29 patients with unresectable thymic carcinoma treated with concurrent chemoradiotherapy, and found an overall response rate of 50%, including 25% complete responses and 25% partial responses^28^. No life-threatening side effects were noted in their report, and a conclusion was made that concurrent chemoradiotherapy for thymic carcinoma was effective, safe, and feasible. Liu *et al*. reported their retrospective cohort analysis of 43 patients with incompletely resected stage III and IVa thymomas^27^. On univariate analysis, they found that DS, radiotherapy, and the presence of myasthenia gravis were associated with better survival. After adjustment with multivariate analysis, radiotherapy was found to be independently associated with a better survival. The above studies have shown that radiotherapy is effective for incomplete or unresectable thymic malignancies. However, these studies included patients with stage IVa thymoma as well as patients with thymic carcinoma, and the radiation doses used were not uniform. The role of dRT in the treatment of unresectable Masaoka-Koga stage III thymoma was not properly evaluated. In the current study, following the ITMIG recommendations, radiotherapy was divided into dRT and non-dRT according to the treatment intent and dose delivered, and the role of dRT in survival and tumor control was analyzed in a more targeted and purposeful manner. Though no clear dose response relationship was found for patients who received dRT, the benefits of dRT for OS, PFS, and FFLF were well demonstrated in the current study, which provides good evidence for the clinical use of dRT for unresectable stage III thymoma.

The most important prognostic factor for thymoma is complete resection, which is also the first choice of treatment for patients with thymoma without metastasis. Whether or not DS should be carried out for locally advanced, Masaoka-Koga stage III thymoma remains controversial. Some authors have reported that compared with biopsy alone, DS improves OS^3,5–11^, while others have reported similar survival rates for the two treatments^1,2,12–20^. A meta-analysis of published retrospective cohort studies was performed to acquire higher-level evidence for the role of DS^32^. Although the results suggested that DS for unresectable thymoma may be associated with improved OS over treatment with surgical biopsy alone, the meta-analysis was limited by the existence of significant heterogeneity between the included studies^32^. Therefore, there is still no high-level evidence supporting the use of DS for the treatment of thymoma. In our study, multivariate analysis with adjustments for confounding factors showed that DS provided no survival benefit nor did it increase tumor-control rates. A systematic review of the treatment of all stages of thymoma has published guidelines on the role of surgery, and has indicated that DS is not recommended as the initial step in the management of unresectable stage III thymomas^4^. However, DS followed by dRT may also be offered to patients with very huge tumors. After DS, the tumor size is minimized and the compression of adjacent tissue is relieved, which could result in less damage to the adjacent tissue during radiotherapy due to a reduced radiation target volume.

In addition to radiotherapy, our study found that tumor size ≤7 cm was associated with improved OS, PFS, FFLF, and FFDM, which implies that tumor size may be an important prognostic predictor for incompletely resected or unresectable thymoma. Roden *et al*. reported similar results, that tumor size was independently associated with OS and disease-free survival (DFS)^33^. Yamada *et al*. reported that tumor size was an adverse factor for PFS in patients with incompletely resected stage III thymoma^34^. However, the IGMIT database and tumor, node, metastasis (TNM) staging system did not found tumor size to be an independent prognostic factor for patients with complete resection^21,35,36^. The prognostic value of histopathological subtype is also under debate, as previously reported results were conflicting. Some studies have shown that the B2 and B3 subtypes are associated with worse OS, while others have found no significant difference in OS between subtype B3 thymoma and other subtypes^37–40^. Our results showed that the B2/B3 subtypes was an independent prognostic factor for worse OS and FFDM. We also evaluated the prognostic value of each subtype separately in the univariate analysis and found that the B3 subtype (P = 0.009) as well as the B2/B3 subtypes together (P = 0.002) were associated with significantly worse OS when compared with other subtypes. However, after multivariate analysis, only the B2/B3 subtypes together showed independent prognostic significance. Great-vessel invasion was found to be associated with lower FFLF and FFDM in the current study. It is difficult to achieve good tumor control when the great vessels have been invaded, especially if multiorgan invasion exists. A Japanese retrospective study reported that patients with great-vessel invasion had lower resectability rates than those without great-vessel invasion, and a higher number of involved organs was associated with worse disease-specific survival^34^.

There are some shortcomings of the current study. First, owing to the retrospective nature of our study, case-selection bias was inevitable. Second, because of the low incidence and indolent biological behavior of thymomas, the study spanned a large period of time to acquire the largest possible sample size. Even so, our results showed that treatment decade had no significant effect on therapeutic efficacy. Third, the sample size of this study may not be well powered to perform multivariate analysis, but many more cases are needed for propensity score matching analysis. To adjust for bias due to confounding factors, we believe that a multivariate analysis is the most suitable statistical method in this study.

## Conclusions

This is the largest known study with detailed analysis to evaluate the role of dRT and DS for unresectable Masaoka-Koga stage III thymoma. The results showed that dRT significantly improved both the survival rate and the treatment-failure rate as compared with the non-dRT group. Multivariate analysis showed that dRT was an independent predictor of OS, PFS, and FFLF. Tumor size was a significant prognostic factor for OS, PFS, FFLF, and FFDM. Histological subtype B2/B3 was an independent risk factor for worse OS and FFDM. Great-vessel invasion was independently associated with worse FFLF and FFDM. Compared with biopsy only, DS did not provide any survival benefit nor reduce treatment-failure rates. To further investigate the role of dRT in this disease, prospective multicenter trials should be performed.

## Data Availability

The datasets used during the current study are available from the corresponding author on reasonable request.
